# Hoechst 33342 Is a Hidden “Janus” amongst Substrates for the Multidrug Efflux Pump LmrP

**DOI:** 10.1371/journal.pone.0141991

**Published:** 2015-11-05

**Authors:** Arthur Neuberger, Hendrik W. van Veen

**Affiliations:** Department of Pharmacology, University of Cambridge, Cambridge, United Kingdom; Hungarian Academy of Sciences, HUNGARY

## Abstract

Multidrug transporters mediate the active extrusion of antibiotics and toxic ions from the cell. This reaction is thought to be based on a switch of the transporter between two conformational states, one in which the interior substrate binding cavity is available for substrate binding at the inside of the cell, and another in which the cavity is exposed to the outside of the cell to enable substrate release. Consistent with this model, cysteine cross-linking studies with the Major Facilitator Superfamily drug/proton antiporter LmrP from *Lactococcus lactis* demonstrated binding of transported benzalkonium to LmrP in its inward-facing state. The fluorescent dye Hoechst 33342 is a substrate for many multidrug transporters and is extruded by efflux pumps in microbial and mammalian cells. Surprisingly, and in contrast to other multidrug transporters, LmrP was found to actively accumulate, rather than extrude, Hoechst 33342 in lactococcal cells. Consistent with this observation, LmrP expression was associated with cellular sensitivity, rather than resistance to Hoechst 33342. Thus, we discovered a hidden “Janus” amongst LmrP substrates that is translocated in reverse direction across the membrane by binding to outward-facing LmrP followed by release from inward-facing LmrP. These findings are in agreement with distance measurements by electron paramagnetic resonance in which Hoechst 33342 binding was found to stabilize LmrP in its outward-facing conformation. Our data have important implications for the use of multidrug exporters in selective targeting of “Hoechst 33342-like” drugs to cells and tissues in which these transporters are expressed.

## Introduction

Drug transporters play a vital role in the intrinsic and acquired resistance of microorganisms against antibiotics and cytotoxic agents [1–3]. Some of these drug transporters are rather specific for a drug or class of drugs, whereas others recognize a wide range of structurally diverse substrates. Bacterial multidrug transporters fall into 5 protein families of which the Major Facilitator Superfamily (MFS) is the largest [4]. The 408-amino-acid MFS member LmrP from *Lactococcus lactis* functions as a drug-proton antiporter that utilizes both the membrane potential (interior negative) and the chemical proton gradient (interior alkaline) of the proton-motive force to mediate the efflux of amphiphilic substrates from the cell [5–8]. Evidence for the proton-motive force-dependent transport of drugs, independent of any other accessory proteins, was provided in studies in proteoliposomes containing purified and functionally reconstituted protein in which ion gradients were imposed artificially [9,10].

One typical LmrP substrate is the fluorescent dye Hoechst 33342. In aqueous solution, Hoechst 33342 is essentially non-fluorescent, whereas its partitioning in the membrane and binding to DNA are associated with a remarkable enhancement of the fluorescence intensity [11]. LmrP-mediated Hoechst 33342 transport has mostly been studied in inside-out membrane vesicles in which transport from the membrane to the aqueous buffer is associated with a decrease in fluorescence [12]. The transport reaction in membrane vesicles is inhibited by other LmrP substrates: competitively in the presence of quinine and verapamil, non-competitively by nicardipin and vinblastin, and un-competitively by tetraphenylphosphonium ions [12]. This finding suggested that LmrP has multiple drug binding sites, which would explain its ability to transport drugs belonging to different classes of antibiotics including lincosamides, macrolides, streptogramins, and tetracyclines [13]. LmrP is predicted to contain a large internal substrate-binding cavity that contains catalytic carboxylates on its surface: D235 and E327 are located in the apex, and D142 is located at a side [6]. All three carboxylates can function as proton binding sites, whereas D235 and E327 have also been implicated in the binding of the divalent cationic substrates Ca^2+^ and propidium [7,9]. Residues Asp 128 and Asp 68, located in cytosolic loops outside the internal cavity were also shown to be involved in the proton motive force-mediated change of LmrP’s accessibility for substrates [14].

Using cysteine cross-linking techniques with a cysteine-less LmrP mutant containing I34C in the N-terminal half and V240C in the C-terminal half, evidence was obtained that the protein can switch between at least two major conformational states that are interconverted by the movement of both halves of the transporter, i.e., the inward-facing conformation in which I34C and V240C are in close proximity and can interact with each other, and the outward-facing conformation in which these cysteines are too far apart for cross-linking [8]. Using this method, it was found that the binding of the transport substrate benzalkonium to LmrP stabilizes the protein in the inward-facing conformation in which the binding cavity is opened to the inside of the cell. However, more recently, measurements of intramolecular distances in LmrP by electron paramagnetic resonance (EPR) techniques suggested that the binding of Hoechst 33342 to LmrP stabilizes the protein in the outward-facing conformation, and questioned the relevance of an inward-facing conformation for drug binding in the transport cycle of LmrP [15].

The paradigm in these and related studies has always been that fluorescent dyes such as ethidium, propidium and Hoechst 33342 are expelled from the cell by LmrP by active efflux. Here, we studied Hoechst 33342 transport by LmrP in more detail, and conclude that the direction of transport is reversed in cells compared to that of other dyes. The active uptake of Hoechst 33342 provides a connecting explanation for the recent hypotheses on LmrP’s transport cycle.

## Methods

### Bacterial strains, plasmids, and growth conditions

*Lactococcus lactis* strain NZ9000 Δ*lmrA* Δ*lmrCD* [16] harboring the expression vector pNZ8048 [17] without insert (control) or with the *lmrP* gene (pHLP5) [5] or *lmrCD* genes [18] inserted downstream of the nisin A-inducible promoter [17] were grown at 30°C in M17 broth (Oxoid Ltd., Basingstoke, UK) supplemented with 25 mM glucose and 5 μg/mL chloramphenicol. Cells grown in overnight (< 16 h) cultures were transferred into fresh medium (1:25 v/v dilution) after which 10 pg/mL nisin A (0.1% v/v of the culture supernatant of nisin A-producing *L*. *lactis* strain NZ9700 [17]) was added when the OD_660_ had reached 0.55 [8]. After 2 h of nisin-induced protein expression, cells were harvested by centrifugation at 3030 g for 10 min at 4°C.

### Hoechst 33342 and ethidium transport in intact cells

**Fig 1:**
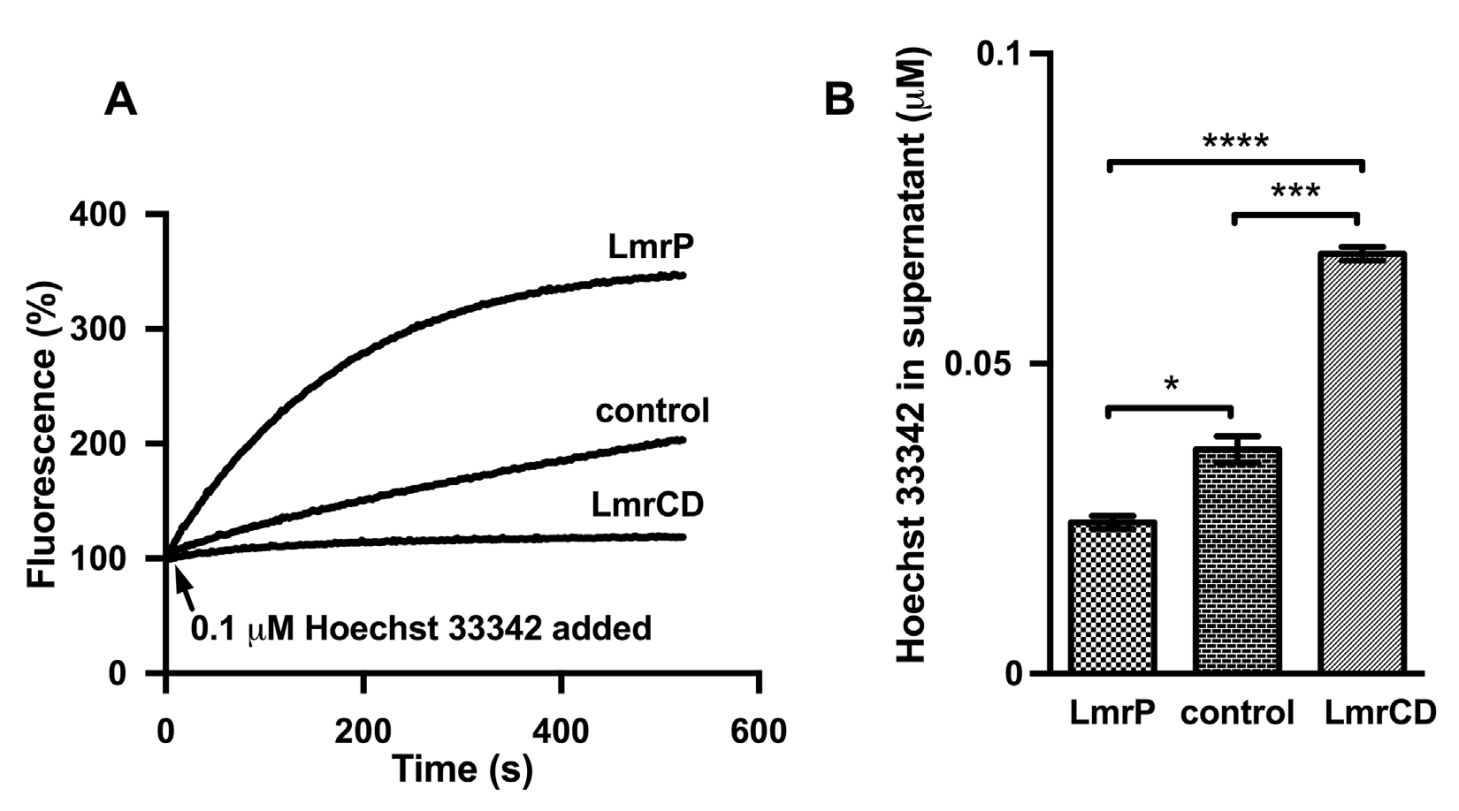
LmrP accumulates Hoechst 33342 in cells, whereas LmrCD disperses Hoechst 33342 in the extracellular buffer. (A) Lactococcal cells expressing LmrP, LmrCD, or neither of these proteins (control) were first allowed to generate metabolic energy for 3 min through the addition of 25 mM glucose before Hoechst 33342 was added at a final concentration of 0.1 μM. Hoechst 33342 transport over time was followed by fluorimetry. Fluorescence traces represent data obtained in three independent experiments using different batches of cells. (B) The concentration of Hoechst 33342 in the supernatant of the cell suspensions in (A) at 540 s was measured in triplicate after addition of 1 mg/mL calf thymus DNA, and is presented as mean ± SEM. (*, p < 0.05; ***; p < 0.001; ****, p < 0.0001).

**Fig 2:**
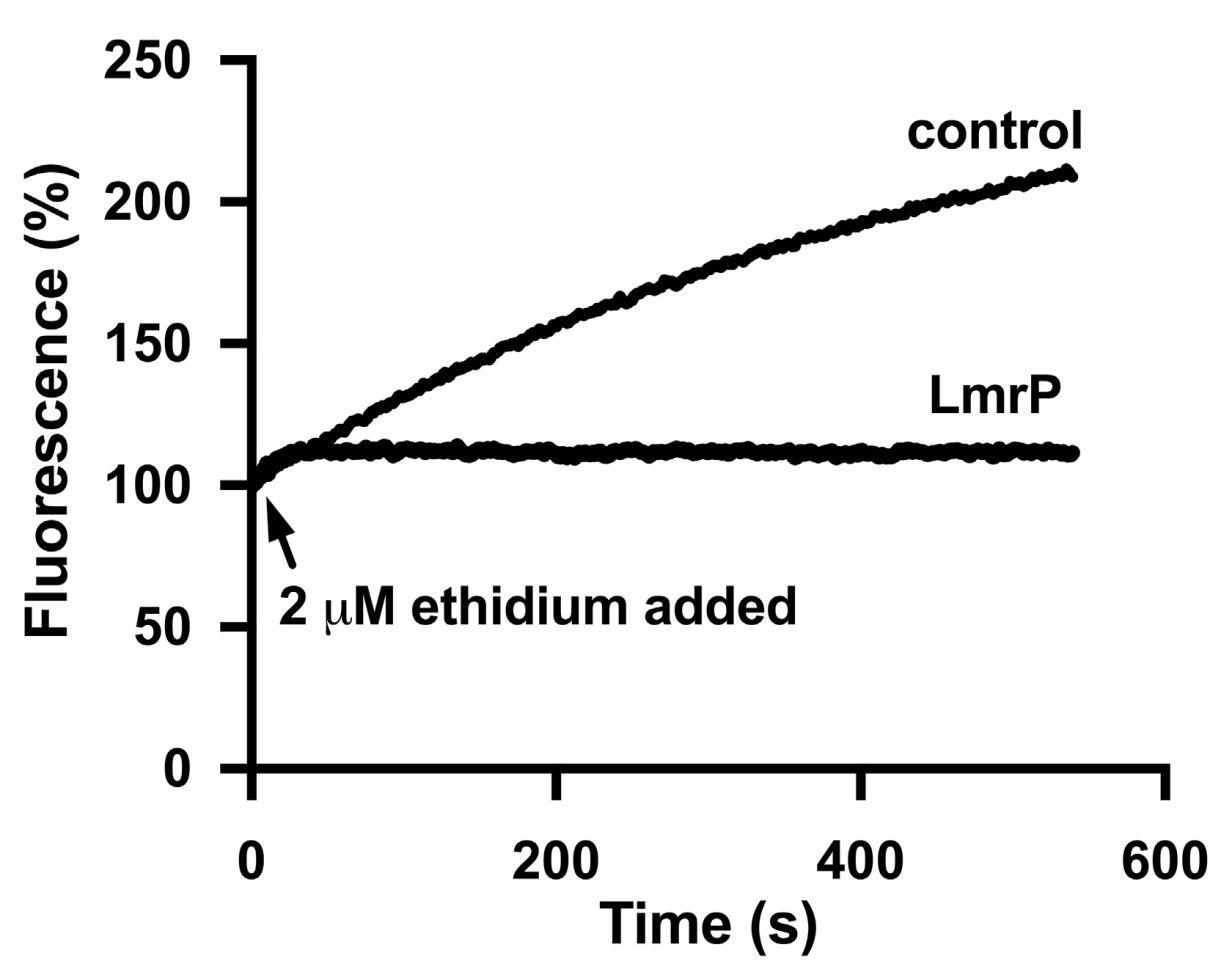
LmrP expressing cells show reduced accumulation of ethidium compared to control cells. The substrate accumulation experiments described in Fig 1A were performed with ethidium at a final concentration of 2 μM. Traces represent data obtained in three independent experiments using different batches of cells.

For measurements of substrate accumulation in cells that generate metabolic energy (Figs 1A and 2), cells were washed 3 times in ice-cold 50 mM KPi, 5 mM MgSO_4_ (pH 7.0) and re-suspended in this buffer to an OD_660_ of 5.0, and kept on ice. In the transport experiments, the cell suspensions were diluted in the KPi buffer at 30°C. Glucose (25 mM) was added 3 min before the addition of 0.1 μM Hoechst 33342 (trihydrochloride salt, fluoropure grade, Molecular Probes, Thermofisher Scientific) or 2 μM ethidium (bromide salt, molecular grade, Promega). Dye accumulation was monitored by fluorimetry in a Perkin Elmer LS-55B fluorescence spectrometer. Hoechst 33342 fluorescence was measured at excitation and emission wavelengths of 355 and 457 nm, respectively, with slit widths of 5 and 10 nm, respectively. For ethidium fluorescence, the excitation and emission wavelengths were set to 500 and 580 nm, respectively, with slit widths to 5 nm and 10 nm, respectively.

The fluorescence emission of Hoechst 33342 in cells is sensitive to environmental parameters, such as intracellular pH, membrane potential, accessibility of chromosomal DNA and plasma membrane to Hoechst 33342 binding, and many others. We therefore also measured the concentration of free Hoechst 33342 in the culture supernatant using standardized conditions. Cells were spun down 9 min after addition of Hoechst 33342 to measure the concentration of the free dye in the supernatant in a Hoechst 33342-to-DNA binding assay (Fig 1B). The supernatant was transferred into a sterile tube, to which calf thymus DNA (Trevigen Inc.) was added to a final concentration of 1 mg/mL. Aliquots (in triplicate) were transferred into wells of a sterile black 96-well plate. Fluorescence of the Hoechst 33342-DNA complex was measured in a Spectramax Gemini XS plate reader (with excitation and emission wavelengths of 355 and 457 nm, respectively) and compared with the fluorescence of Hoechst 33342 standards in a calibration curve. For the calibration curve, calf thymus DNA was diluted in 50 mM KPi (pH 7.0) supplemented with 5 mM MgSO_4_ and 25 mM glucose at 20°C. Hoechst 33342 was added at a final concentration of 0, 0.025, 0.05, 0.075 and 0.1 μM. Aliquots of triplicates of these dilutions were transferred to wells of a 96-well plate. Data were fitted by linear regression.

**Fig 3:**
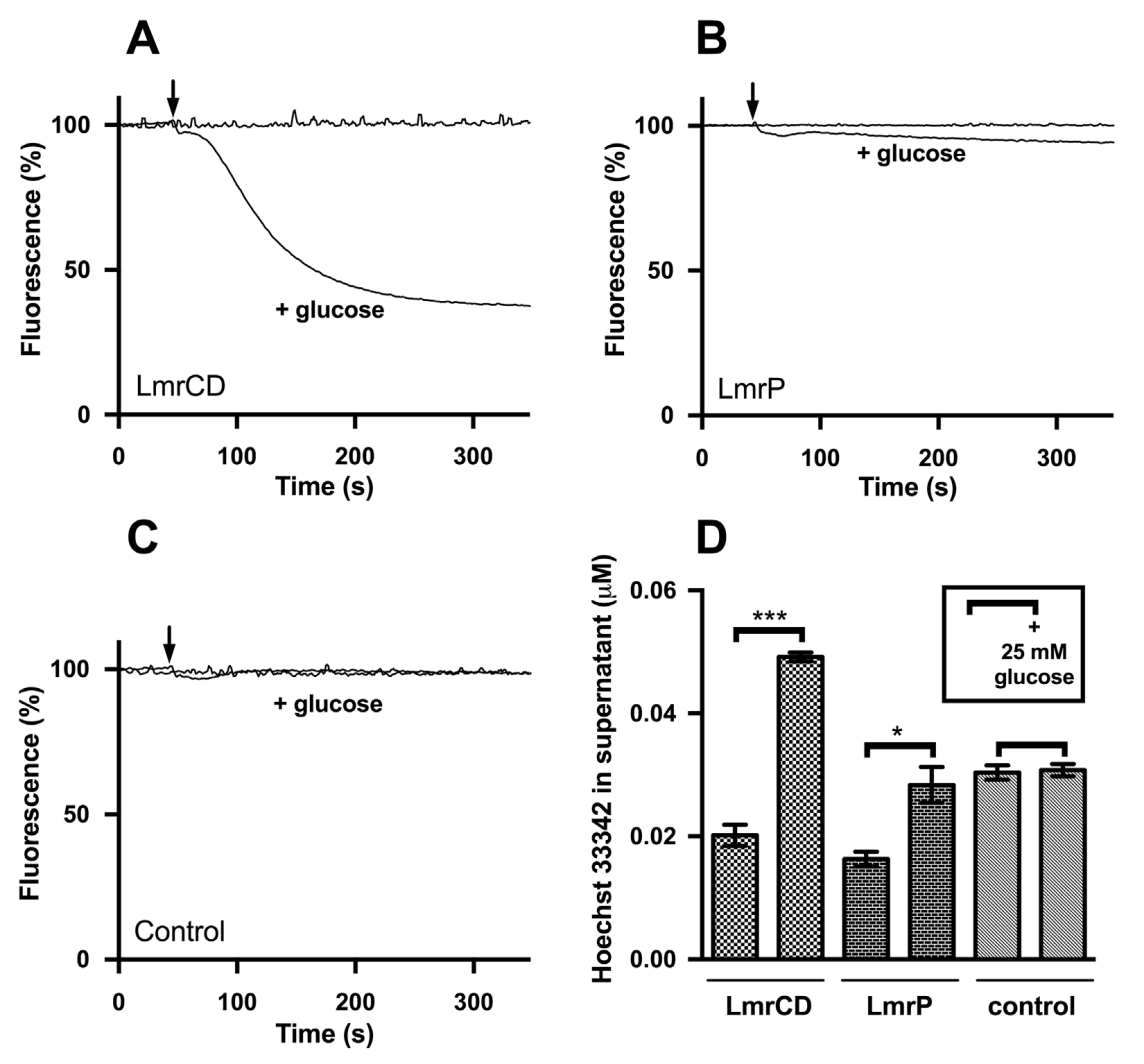
Active Hoechst 33342 efflux in cells is significant for LmrCD but weak for LmrP. (A-C) ATP-depleted cells with LmrCD (A), LmrP (B) or without LmrCD or LmrP proteins (control) (C) were preloaded with Hoechst 33342 at a final concentration of 0.1 μM. At the arrow, 25 mM glucose was added to one aliquot of the cells whereas in the other aliquot the cells remained de-energized. Traces are typical for data obtained in three independent experiments using different batches of cells. (D) The concentration of Hoechst 33342 (mean ± SEM, n = 3) in the supernatant of the cell suspensions in (A-C) at 900 s was measured after addition of 1 mg/mL calf thymus DNA. (*, p < 0.05; ***; p < 0.001).

**Fig 4:**
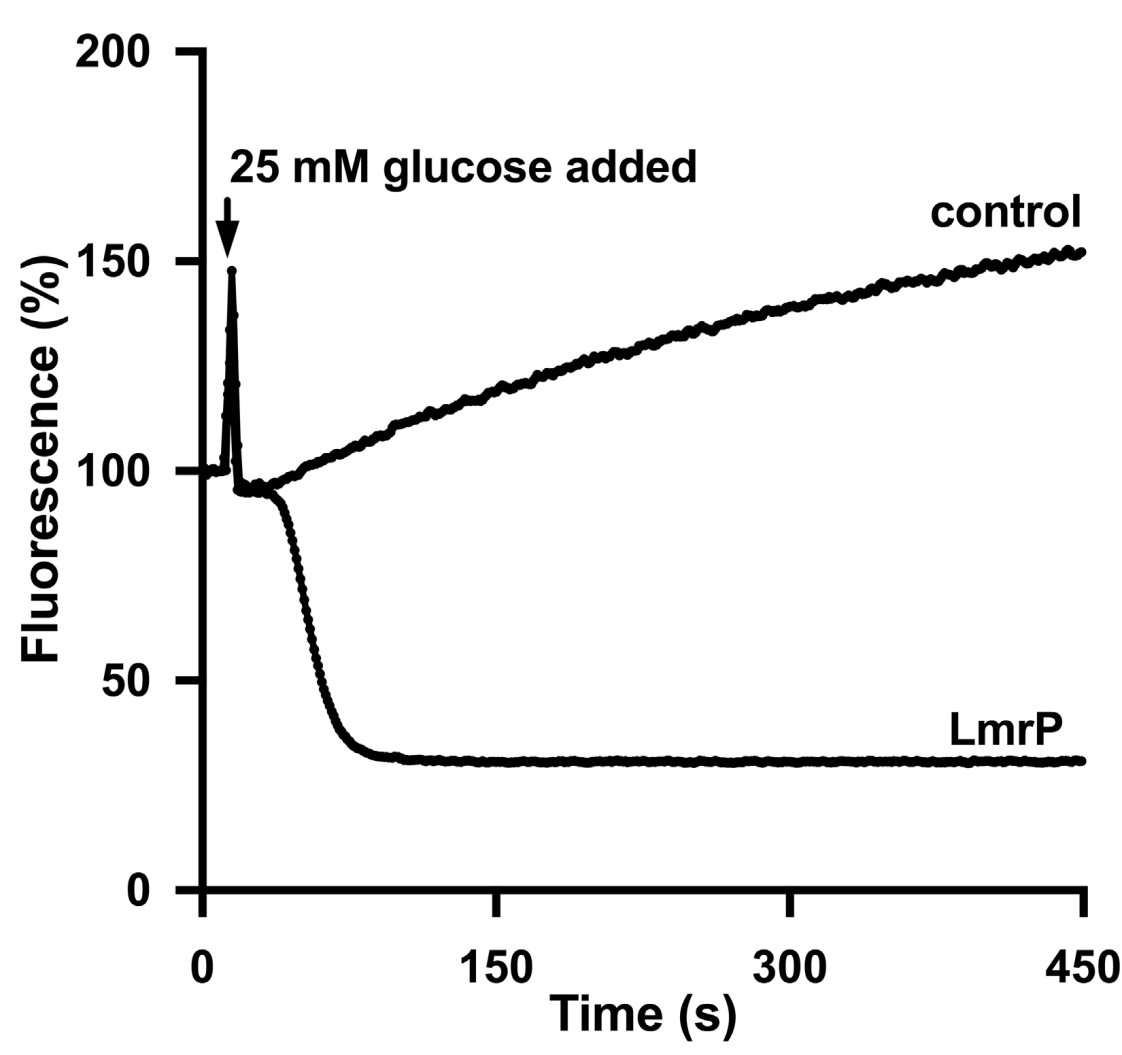
LmrP mediates active efflux of ethidium. The efflux experiments described in Fig 3 were performed with ethidium at a final concentration of 2 μM. Traces represent data obtained in three independent experiments using different batches of cells.

To measure dye efflux from preloaded cells (Figs 3A–3C and 4), cells were first ATP-depleted by washing once with 50 mM KPi (pH 7.0) supplemented with 5 mM MgSO_4_, and resuspending in the same KPi buffer containing 0.5 mM 2,4-dinitrophenol (DNP) [19]. Following incubation for 30 min at 30°C, cells were subsequently washed 3 times with ice-cold 50 mM KPi buffer (pH 7.0) containing 5 mM MgSO_4_, and re-suspended in this buffer to an OD_660_ of 5.0 and kept on ice. Cells were pre-loaded at 30°C with 0.1 μM Hoechst 33342 or 2 μM ethidium until the fluorescence emission reached a constant level. Glucose (25 mM) was then added as a source of metabolic energy to assess active substrate efflux. After 15 min of incubation in the presence of glucose, the cells were spun down in an Eppendorf 5415 D centrifuge at 16,168g for 5 min to measure the free Hoechst 33342 concentration in the culture supernatant (Fig 3D).

All Hoechst 33342 and ethidium transport experiments were performed in triplicate. Figs 1–4 contain representative traces of 3 or more independent experiments using separate batches of cells. Fluorescence data are normalized to the fluorescence start level at t = 0 s to allow direct comparisons between different strains. Hoechst 33342 concentrations in the supernatant were measured in triplicate, and are presented as mean ± SEM. Significance was tested using Welch's (unpaired) t-test.

### Hoechst 33342 sensitivity of cells

**Fig 5:**
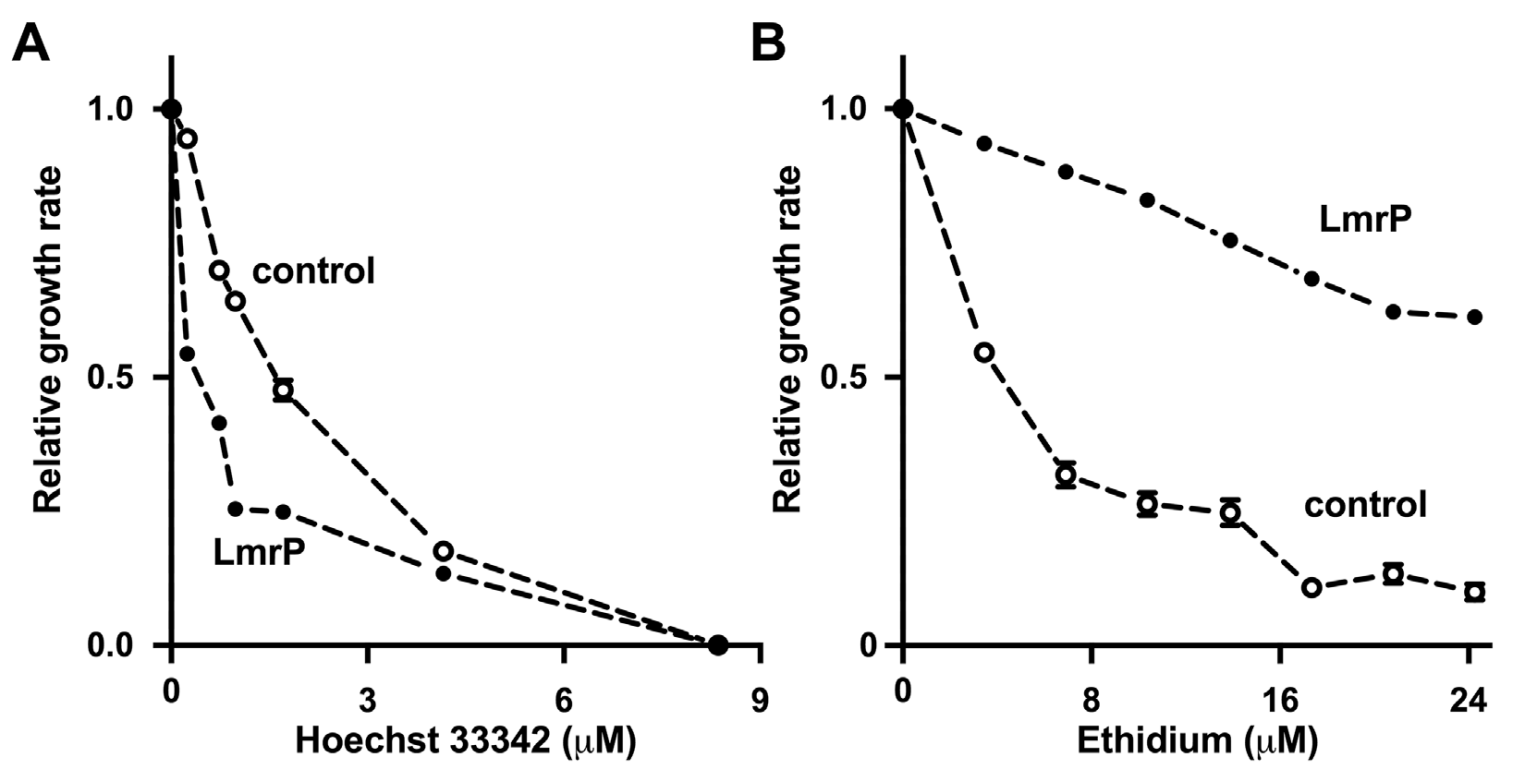
LmrP mediates cellular sensitivity to Hoechst 33342 but resistance to ethidium. Growth rate of LmrP-expressing lactococcal cells and cells without LmrP expression (control) in the presence of up to 8.4 μM Hoechst 33342 (A), or up to 24 μM ethidium (B) was determined relative to the maximum growth rate in the absence of drug. The error bars for some of the data points were too small to be displayed, and are hidden behind the data point symbols.

Overnight cultures from glycerol stocks of *L*. *lactis* strain NZ9000 Δ*lmrA* Δ*lmrCD* harboring the expression vector pNZ8048 without insert (control) and with the *lmrP* gene (pHLP5) were grown in M17 containing 25 mM glucose and 5 μg/mL chloramphenicol. Cultures were grown for about 16 h at 30°C, and then used to inoculate fresh medium for protein production through the addition of 10 pg/mL nisin at OD_660_ of 0.55. After 2 h of induction, the cells were diluted ten-fold in fresh medium containing 10 pg/mL nisin A in the wells of a 96-well plate after which Hoechst 33342 or ethidium was added as indicated in Fig 5. Growth was followed over time at OD_660_ at 30°C in a Versamax microplate reader (Molecular Devices). For the determination of the relative growth rates of the cultures in each of the wells, the exponential phase of the growth curve was determined from the linear increase in a ^10^log(OD_660_) versus time plot. The slope of this section was determined by simple linear regression. Heteroscedasticity-consistent standard errors of the corresponding slope coefficient were calculated. The quality of the fit was significant in all cases (P < 0.05). Next, the relative growth rate was determined as the ratio of the growth rate in the presence of drug over the maximum growth rate in the absence of drug. The drug concentration that caused a 50% reduction in the relative growth rate (IC_50_) was then determined from a plot of the relative growth rate versus drug concentration (Fig 5).

## Results

Hoechst 33342 transport was measured in intact cells of *L*. *lactis* NZ9000 Δ*lmrA* Δ*lmrCD* expressing LmrP or LmrCD via the NICE expression system [17], and in cells without expression of these multidrug transporters. Upon addition of Hoechst 33342 at a final concentration of 0.1 μM to cells in which metabolic energy was generated from glucose catabolism, dye accumulation in LmrCD expressing cells was low compared to control cells indicating active LmrCD-mediated Hoechst 33342 efflux (Fig 1A). Due to its amphiphilic properties, Hoechst 33342 partitions easily into the cytoplasmic bilayer and from there over time to the inside of the cell where it binds to DNA with a concomitant fluorescence intensity enhancement [11]. The modestly increasing fluorescence in control cells therefore served as the baseline for passive Hoechst 33342 influx in the cells. However, the much more rapid and overall higher accumulation of Hoechst 33342 in LmrP expressing cells clearly indicated active LmrP-dependent influx of the dye.

The fluorescence emission of Hoechst 33342 is greatly enhanced by the partitioning of the dye in lipid membranes and by its binding to the minor groove in DNA. However, as these interactions can be altered by protonation and/or deprotonation of the bis-benzimidazole structure at 5 nitrogen atoms, the level of fluorescence emission by the dye in cells is a multifactorial parameter [20]. Therefore, the accumulation of Hoechst 33342 in cells was also evaluated in a complementary assay based on the measurement under standardized conditions of the remaining Hoechst 33342 concentration in the culture supernatant (Fig 1B). After 9 min of incubation with 0.1 μM Hoechst 33342, cells were spun down and aliquots of supernatant were mixed with calf thymus DNA. Fluorescence of the Hoechst 33342-DNA complex was compared with that of Hoechst 33342 standards. The measurements revealed significant differences in the concentrations of free Hoechst 33342 in the cell suspensions. The concentration of Hoechst 33342 was the highest in the supernatant of LmrCD expressing cells, which is consistent with the efflux of Hoechst 33342 from these cells. A lower concentration was measured in the supernatant of control cells, in line with the absence of LmrCD activity. Remarkably, a significantly lower concentration than control was found in the supernatant of LmrP expressing cells, consistent with the enhanced accumulation of the dye in cells containing LmrP (Fig 1A). Both the measurements of transport and extracellular Hoechst concentrations therefore suggest active LmrP-mediated influx of Hoechst 33342 into lactococcal cells. The transport activity of LmrP was also tested in the same cell suspensions in a robust ethidium transport assay that is based on ethidium intercalation in intracellular nucleic acids (Fig 2). Upon addition of 2 μM ethidium, dye accumulation remained low for LmrP expressing cells whereas control cells rapidly accumulated ethidium over time. Consistent with previous publications [6,7], these data demonstrate LmrP’s ability to catalyze ethidium extrusion.

The ability of LmrP to mediate Hoechst 33342 efflux was tested in ATP-depleted cells in which Hoechst 33342 was first allowed to accumulate until a steady-state level was reached. Next, 25 mM glucose was added to the cells to allow the generation of metabolic energy to activate LmrP activity. The subsequent decrease in Hoechst 33342 fluorescence was most pronounced for LmrCD expressing cells (Fig 3A) but was very weak for LmrP expressing cells (Fig 3B) compared to the control (Fig 3C). The free Hoechst 33342 concentration in the supernatant was quantified after a prolonged incubation of 15 min after glucose addition when no further decrease in fluorescence could be observed. The same was done for cells without glucose addition for exactly the same amount of time. A comparison of measured Hoechst 33342 concentrations in the supernatant suggested active Hoechst 33342 efflux from LmrP and LmrCD expressing cells with a significantly greater efflux activity for the latter. The concentration of Hoechst 33342 in the supernatant of control cells was indistinguishable in the presence or absence of glucose (Fig 3D). The functionality of LmrP in the ATP-depleted cells was also tested using ethidium as the substrate. The addition of glucose elicited a robust ethidium efflux from the ethidium-loaded cells containing LmrP whereas control cells showed an increase in ethidium fluorescence. The latter can be explained by the stimulation of the passive influx of ethidium in these cells in response to the rise in the membrane potential (interior negative) during glucose metabolism (Fig 4).

Finally, the active influx of Hoechst 33342 by LmrP in cells was also observed in growth experiments in which LmrP expression was associated with a sensitivity for Hoechst 33342 compared to the control (Fig 5A). Here, LmrP expressing cells exhibited an IC_50_ value of 0.39 ± 0.04 μM as compared to 1.60 ± 0.02 μM for control cells. A similar experiment with ethidium demonstrated the LmrP-mediated resistance against this dye (Fig 5B). In this case, an IC_50_ value of 4.20 ± 0.11 μM was determined for the control, whereas the IC_50_ value for LmrP expressing cells exceeded 24 μM. These data show that LmrP expression confers Hoechst 33342 sensitivity on the lactococcal cells, rather than Hoechst 33342 resistance, whereas for ethidium the usual resistance is observed.

## Discussion

The MFS drug/proton antiporter LmrP confers resistance on lactococcal cells to 22 clinically used antibiotics [13]. Many antibiotics are amphiphilic compounds that bind to the membrane-water interface with exposure of their hydrophobic moieties to the phospholipid bilayer and hydrophilic moieties to the aqueous environment [21]. Due to the close alignment of the acyl chains of the phospholipids and the overall hydrophobicity of this region, the membrane provides a barrier that limits the rate of passive influx of ions and antibiotics [22]. The cellular uptake of drugs is facilitated by the presence of cationic moieties that enhance accumulation at the inside surface of the membrane in response to the transmembrane potential (interior negative). Detailed studies have shown that LmrP mediates the transbilayer movement of the amphiphilic cationic substrate TMA-DPH (1-(4-(trimethylamino)phenyl)-6-phenylhexa-1,3,5-triene) from the inner leaflet of the cytoplasmic membrane to the external side of the cell [5], thus effectively facilitating the transbilayer efflux of substrate across a permeability barrier at a rate that exceeds the rate of passive substrate influx.

Based on crystallographic data for secondary-active membrane transporters, including LacY and LeuT, these transporters are thought to operate by an alternating mechanism involving at least an inward-open and outward-open state [23,24] in which conformational transitions are triggered by the binding and dissociation of substrate and coupling ion(s). This alternating access mechanism is also likely to be relevant for LmrP, although the sequence of molecular steps in proton and substrate binding and release, and their relation to the conformational changes that underlie this mechanism, are not well resolved. For LmrP, two different models have been proposed to explain drug efflux. Firstly, cysteine cross-linking studies with LmrP C270S I34C V240C suggest that benzalkonium binding stabilizes a conformation that is inward-facing (Model 1) [8]; a similar working model was also proposed for the *E*. *coli* MFS multidrug transporter MdfA [25]. During proton-drug antiport, carboxylates in LmrP are protonated in the outward-facing conformation. This protonation facilitates a chemical proton gradient-dependent conformational switch to the inward-facing conformation, which allows (and might also require) substrate binding from the interior of the cell. Proton release at the inside surface subsequently facilitates a second conformational switch in which dissociated carboxylates reorient back to outward-facing in a membrane potential (interior negative)-dependent fashion. The cycle is completed by reprotonation of carboxylates at the external side of the membrane with concomitant substrate release [8]. Secondly, a previous study on LmrP based on distance measurements by EPR suggested that Hoechst 33342 binds from the cellular interior to the outward-facing state, rather than the inward-facing state, and proposed that subsequent proton binding coordinates substrate release from this state into the extracellular environment (Model 2) [15]. Proton movement via acidic residues eventually leads to protonation of Asp68 at the intracellular side of LmrP, which induces closure of LmrP on the extracellular side with concomitant opening on the intracellular side. This opening is thought to lead to (partial) exposure to the intracellular milieu, allowing for deprotonation of Asp68, at which point LmrP is thought to re-set to the resting (occluded) state [15]. A new cycle of Hoechst 33342 binding to the outward-facing state can now be initiated.

These two models were developed using experimental procedures that differed methodologically in the choice of substrates. Whereas the studies leading to Model 1 used benzalkonium and ethidium as substrates that are known to be effluxed by LmrP via substrate-proton antiport [8], Model 2 was based on the interaction of LmrP with Hoechst 33342. Our study shows that, in fact, the addition of Hoechst 33342 to the external side of LmrP-expressing cells results in active influx of Hoechst 33342. The stabilization of an outward-facing state by Hoechst 33342 but inward-facing state by benzalkonium can now be explained in the light of LmrP’s opposite direction of transport for these substrates. For each of these, the substrate initially binds to the transporter at the side of the membrane from where transport is initiated. The uptake of Hoechst 33342 by LmrP is unexpected, as LmrCD and other multidrug transporters mediate the efflux of both substrates (see Fig 1). In our experiments, a weak LmrP-mediated efflux of Hoechst 33342 was only observed after a substantial intracellular pool of the dye was generated through preloading of the cells (Fig 3B). Our experiments on LmrP-mediated influx of Hoechst 33342 in cells have implications for earlier observations on LmrP-mediated Hoechst 33342 transport in lactococcal inside-out membrane vesicles [12,13], which might reflect transport of Hoechst 33342 from the membrane to the external buffer rather than the lumen of these membrane vesicles.

Substrate uptake by multidrug transporters is an interesting feature that might be exploited therapeutically in the future to selectively target cells and tissues in which multidrug transporters are upregulated. We previously studied drug uptake by ATP-binding cassette exporters in ATP-depleted cells [19,26]. Our finding of active LmrP-mediated Hoechst 33342 uptake in cells now extends these observations to an MFS multidrug transporter in cells that generate metabolic energy.
